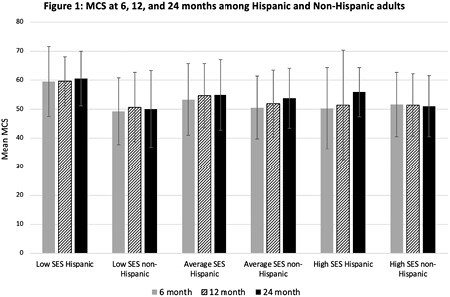# 73 Examining Health-Related Quality of Life in Hispanic Adults with Burn Injuries

**DOI:** 10.1093/jbcr/irae036.065

**Published:** 2024-04-17

**Authors:** Tamara Alcala Dominguez, Kara McMullen, Caitlin M Orton, Haig A Yenikomshian, Elizabeth Flores, Jeffrey C Schneider, Colleen M Ryan, Barclay T Stewart

**Affiliations:** University of Washington, Seattle, Washington; UW Medicine Regional Burn Center, Harborview Medical Center, Seattle, WA; University of Southern California, Los Angeles, CA; Division of Plastic Surgery, University of Southern California Keck School of Medicine, Los Angeles, CA; Spaulding Rehabilitation Hospital/Harvard Medical School, Boston, MA; Massachusetts General Hospital/Shriners Children's, Boston, MA; University of Washington, Seattle, Washington; UW Medicine Regional Burn Center, Harborview Medical Center, Seattle, WA; University of Southern California, Los Angeles, CA; Division of Plastic Surgery, University of Southern California Keck School of Medicine, Los Angeles, CA; Spaulding Rehabilitation Hospital/Harvard Medical School, Boston, MA; Massachusetts General Hospital/Shriners Children's, Boston, MA; University of Washington, Seattle, Washington; UW Medicine Regional Burn Center, Harborview Medical Center, Seattle, WA; University of Southern California, Los Angeles, CA; Division of Plastic Surgery, University of Southern California Keck School of Medicine, Los Angeles, CA; Spaulding Rehabilitation Hospital/Harvard Medical School, Boston, MA; Massachusetts General Hospital/Shriners Children's, Boston, MA; University of Washington, Seattle, Washington; UW Medicine Regional Burn Center, Harborview Medical Center, Seattle, WA; University of Southern California, Los Angeles, CA; Division of Plastic Surgery, University of Southern California Keck School of Medicine, Los Angeles, CA; Spaulding Rehabilitation Hospital/Harvard Medical School, Boston, MA; Massachusetts General Hospital/Shriners Children's, Boston, MA; University of Washington, Seattle, Washington; UW Medicine Regional Burn Center, Harborview Medical Center, Seattle, WA; University of Southern California, Los Angeles, CA; Division of Plastic Surgery, University of Southern California Keck School of Medicine, Los Angeles, CA; Spaulding Rehabilitation Hospital/Harvard Medical School, Boston, MA; Massachusetts General Hospital/Shriners Children's, Boston, MA; University of Washington, Seattle, Washington; UW Medicine Regional Burn Center, Harborview Medical Center, Seattle, WA; University of Southern California, Los Angeles, CA; Division of Plastic Surgery, University of Southern California Keck School of Medicine, Los Angeles, CA; Spaulding Rehabilitation Hospital/Harvard Medical School, Boston, MA; Massachusetts General Hospital/Shriners Children's, Boston, MA; University of Washington, Seattle, Washington; UW Medicine Regional Burn Center, Harborview Medical Center, Seattle, WA; University of Southern California, Los Angeles, CA; Division of Plastic Surgery, University of Southern California Keck School of Medicine, Los Angeles, CA; Spaulding Rehabilitation Hospital/Harvard Medical School, Boston, MA; Massachusetts General Hospital/Shriners Children's, Boston, MA; University of Washington, Seattle, Washington; UW Medicine Regional Burn Center, Harborview Medical Center, Seattle, WA; University of Southern California, Los Angeles, CA; Division of Plastic Surgery, University of Southern California Keck School of Medicine, Los Angeles, CA; Spaulding Rehabilitation Hospital/Harvard Medical School, Boston, MA; Massachusetts General Hospital/Shriners Children's, Boston, MA

## Abstract

**Introduction:**

The intersectionality of an individual’s social and political position shapes their lived experience and can be associated with inequities. Thus, historically ethnicity has been used as a contextual indicator of inequity and often as a proxy of economic disadvantage. A resulting presumption is that the interplay of ethnic identity and socioeconomic (SES) factors can compound the burden faced by individuals during recovery and affect overall health related quality of life (HRQOL). In this analysis, we hypothesize that Hispanic adults with burn injuries and who had low SES status prior to injury reported decreased HRQOL post-injury compared to those with average-SES.

**Methods:**

Data from adult participants in a national multicenter longitudinal database was analyzed. Ethnicity was provided by self-report or medical record. SES was classified as low ( < $49,000 annual income or no high school diploma/GED), average ($50,000-$99,999 annual income or completion of high school/GED), and high (>$99,999 annual income or at least some college or trade school). VR-12 and PROMIS Global-10 measure HRQOL in both physical and mental health domains, through validated physical component scores (PCS) and mental component scores (MCS). Kruskal Wallis tests were used to test differences of PCS and MCS between subsets of Hispanics and non-Hispanics with low, average, and high SES.

**Results:**

Out of 952 participants, 19.5% were Hispanic. The average age was 40.6 years. Nearly half reported less than a high school education (47.3%), 40.3% were high school graduates, and 12.4% had either a vocational degree or some form of higher education. Non-Hispanic participants averaged 47 years of age, with 14.6% reporting less than a high school education. Nearly a third of Hispanic participants experienced low SES (34.4%), 38.7% experienced average SES, and 26.9% experienced high SES. Conversely, non-Hispanic participants experienced low and average SES less commonly (8.1 and 30.9%, respectively) and 61% experienced high SES (all differences p< 0.001). Burn surface area, sex, and PCS were not statistically different between groups. Notably, the low SES Hispanic group consistently reported statistically significantly higher MCS, compared to all other cohorts, at 6-months (59.6, p=0.0008), 12-months (59.7, p=0.001), and 24-months (60.6, p=0.003) post-injury follow-up (Figure 1).

**Conclusions:**

Hispanic adults with low SES reported higher mental health scores, signifying better HRQOL in this domain, than their average SES Hispanic and non-Hispanic counterparts over a 24-month follow-up period.

**Applicability of Research to Practice:**

These results underscore the complexity of burn recovery experiences and could be suggestive of underlying protective factors that should be explored for possible utilization in culturally nuanced approaches to rehabilitation.